# Comparison of sciatic nerve block quality achieved using the anterior and posterior approaches: a randomised trial

**DOI:** 10.1186/s12871-019-0898-0

**Published:** 2019-12-13

**Authors:** Abdulkadir Yektaş, Bedih Balkan

**Affiliations:** 1 Department of Anesthesiology and Reanimation, Republic of Turkey Health Sciences Univercity Diyarbakır Gazi Yaşargil Training and Research Hospital, Kayapınar Mahallesi Diayarbakır, Turkey; 2 Department of Anesthesiology and Reanimation, Republic of Turkey Health Sciences Univercity Istanbul Dr. Sadi Konuk Training and Research Hospital, Istanbul, Turkey

**Keywords:** Anterior sciatic nerve block, Posterior sciatic nerve block, Block quality

## Abstract

**Background:**

The co-administration of sciatic and femoral nerve blocks can provide anaesthesia and analgesia in patients undergoing lower extremity surgeries. Several approaches to achieve sciatic nerve block have been described, including anterior and posterior approaches.

**Methods:**

In total, 58 study patients were randomly assigned to receive either anterior (group A*, n* = 29) or posterior (group P, *n* = 29) sciatic nerve block. Thereafter, the following parameters were determined: sensory and motor block start and end times, time to first fentanyl requirement after blockade but before the start of the operation, time to first fentanyl requirement after the start of the operation, mean fentanyl dose administered after blockade but before the start of the operation, mean fentanyl dose after the start of the operation, time to first diclofenac sodium dose, and total dose of diclofenac sodium required. The trial was retrospectively registered on 11 July 2018.

**Results:**

The time to initiation of sensory block was significantly shorter in group P than in group A (7.70 ± 2.05 min and 12.88 ± 4.87 min, respectively; *p* = 0.01). Group P also had a significantly shorter time to first fentanyl requirement after block but before the start of the operation (00.00 ± 00.00 min for group P and 4.05 ± 7.47 min for group A; *p* < 0.01), significantly higher mean fentanyl dose per patient after block but before the start of the operation (44.03 ± 23.78 μg for group P and 31.20 ± 27.79 μg for group A), significantly longer time to first fentanyl requirement after the start of the operation (16.24 ± 7.13 min for group P and 00.00 ± 00.00 min for group A; *p* = 0.01), and significantly lower mean fentanyl dose per patient after the start of the operation (11.51 ± 2.87 μg for group P and 147.75 ± 22.30 μg for group A). Patient satisfaction (*p* < 0.01), anaesthesia quality (*p* = 0.006), and surgical quality (*p* = 0.047) were significantly higher in group P.

**Conclusions:**

Anterior and posterior approaches can be used to achieve sciatic nerve block in patients undergoing surgery for malleolar fractures. However, better anaesthesia and pain control results can be obtained if analgesia is administered preoperatively in patients with a posterior approach block and after the start of the operation in patients with an anterior approach block.

## Background

The co-administration of sciatic and femoral nerve blocks provides anaesthesia or analgesia in patients undergoing lower extremity surgeries [1–3]. Sciatic nerve block can be applied using either an anterior or a posterior approach [4]. Anterior sciatic nerve blocks are performed with the patient in the supine position, at the same time and from the same region as femoral nerve blocks. Turning the patient to one side is not required. Following application of the tourniquet, without moving the patient, the patient can be transferred to the operating room. However, the sciatic nerve is located deep and behind the femur [5, 6], which complicates administration of the block; thus, an anterior block is considered an advanced nerve block. The posterior approach is technically easier to perform; however, patients with lower limb fractures often experience pain until the block is achieved, because they must be turned sideways to allow the fractured limb to remain on top.

The use of ultrasonography (USG) together with classical techniques generally increases the success rate when administering a peripheral nerve block [4, 7]. USG has been successfully used in both anterior and posterior sciatic nerve blocks, as well as femoral nerve blocks [4]. The concurrent use of USG and a nerve stimulator has been reported to improve the success rate of the block, as well as the quality of the anaesthesia [8–10].

Prior to a posterior sciatic nerve block, patients with medial and lateral malleolar fractures experience pain when placed in a lateral position. Pain after the block is also common, as indicated by the high Visual Analogue Scale (VAS) scores reported, such that the early addition of additional analgesics is often necessary. Here, we compared the quality of sciatic nerve block performed via the anterior and posterior approaches in patients undergoing lower extremity surgeries. The primary outcome was defined based on the mean time to sensory block onset following a sciatic nerve block administered by the anterior or posterior approach. Secondary results were evaluated based on the time to first fentanyl requirement after block but before the start of the operation, time to first fentanyl requirement after the start of the operation, mean fentanyl dose administered after the block but before the start of the operation, mean fentanyl dose after the start of the operation, time of motor and sensory block onset, duration of sciatic and femoral block, time to first diclofenac sodium administration, total dose of diclofenac sodium administered in the first 24 h postoperatively, postoperative VAS score, patient satisfaction and the quality of anaesthesia and surgery as evaluated by the anaesthesiologist and surgeon respectively.

## Methods

### Patients

**Fig. 1:**
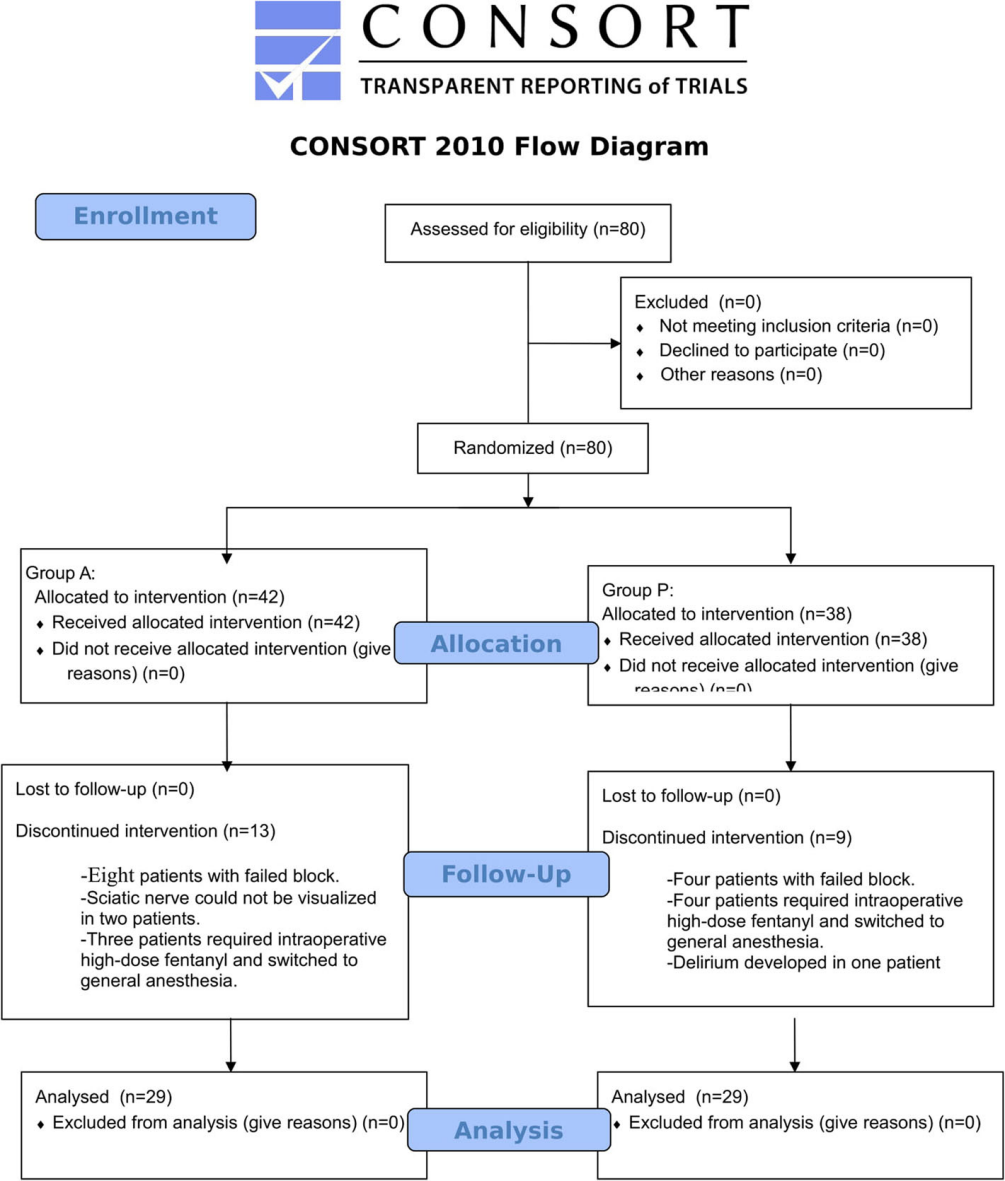
CONSORT 2010 Flow Diagram

The local Ethics Committee approved the study protocol (approval date: 18 March 2013, approval no. 2013/125). This study was conducted between 18 March 2013 and 24 June 2016 in the Surgery Department of the Health Science University of Bagcilar Training and Research Hospital (Turkey), and was performed in accordance with the principles of the Declaration of Helsinki. Written informed consent was obtained from each patient for inclusion in the study. Patients in this single-blinded, prospectively planned study were randomly assigned to anterior or posterior sciatic nerve block. At the beginning of the study, blockage failed in 80 patients, who were subsequently assigned to general anaesthesia (Fig. 1) and thus excluded from further study. After the block was performed, the anaesthesiologist followed these general anaesthesia patients but was blinded to the type of block that had been performed. The single-blinded anaesthesiologist performed all posterior or anterior sciatic nerve and femoral nerve blocks, then exited the operating room after their completion; the patient was repositioned prior to beginning the operation. Another anaesthesiologist followed the patient without information regarding the direction of the blockage.

Inclusion criteria were patients aged 18–65 years with American Society of Anesthesiologists (ASA) physical status classes I or II, who had lateral and/or medial malleolus fractures.

Exclusion criteria were failed block, inability to visualise the sciatic nerve, and/or occurrence of delirium. In addition, patients with vascular, cardiac, or metabolic (diabetes mellitus)–renal–hepatic disease, or with neuropathic pain in the lower extremity were excluded, as were patients who were pregnant, had hemodynamic instability, or were currently taking medications likely to cause metabolic acid–base imbalance. Finally, patients with a history of steroid use or allergy, contraindications to regional anaesthesia, and/or alcohol/drug addiction were also excluded, as were those who did not graduate from primary school.

### Procedure

Variables of interest included patient age, sex, height, weight, and American Society of Anesthesiologists physical status; operation time and tourniquet time; time to first fentanyl requirement before surgery and after blockade; time to first fentanyl requirement after surgery; mean fentanyl dose per patient before surgery and after blockade; mean fentanyl dose per patient after surgery; motor and sensory block start and end times after sciatic and femoral block; time to first diclofenac sodium dose; total dose of diclofenac sodium in the first 24 h postoperatively; VAS score; patient satisfaction; anaesthesia quality, as assessed by the anaesthesiologist; and surgical quality, as assessed by the surgeon.

Patient satisfaction was scored as 0: failed, 1: weak, 2: moderate, 3: good, and 4: excellent [11, 12]. Anaesthesia quality (anaesthesiologist) was scored as follows: 1: general anaesthesia was required, 2: complementary analgesic was needed, moderate complaints, 3: no need for complementary analgesia, few complaints, and 4: no complaints [11]. Surgical quality (surgeon) was scored as follows: 1: failed, 2: moderate, 3: good, and 4: excellent [11].

The patients received an explanation of the VAS on the day preceding surgery. No premedication was administered. In the regional block room, routine monitoring was conducted, including electrocardiography, non-invasive arterial sphygmomanometry, and peripheral pulse oximetry. Sciatic nerve blocks using an anterior approach were performed using a Stimuplex® A needle (21G 0.80–150) positioned at 30°, either isolated or in conjunction with a nerve block stimulator (Stimuplex HNS nerve stimulator; BRAUN, Germany) and USG (diagnostic ultrasound system, model SDU 450 XL class-1 type B; Shimadzu Corp., Japan). Local anaesthetic consisted of 15 mL of 0.5% isobaric bupivacaine, 5 mL of 2% lidocaine, and 20 mL of isotonic sodium chloride. In both the anterior and posterior approaches, nerve stimulation was performed with a frequency of 2 Hz and a current of 1 mA. The stimulus intensity was gradually reduced to 0.4 mA when a response was obtained.

**Fig. 2:**
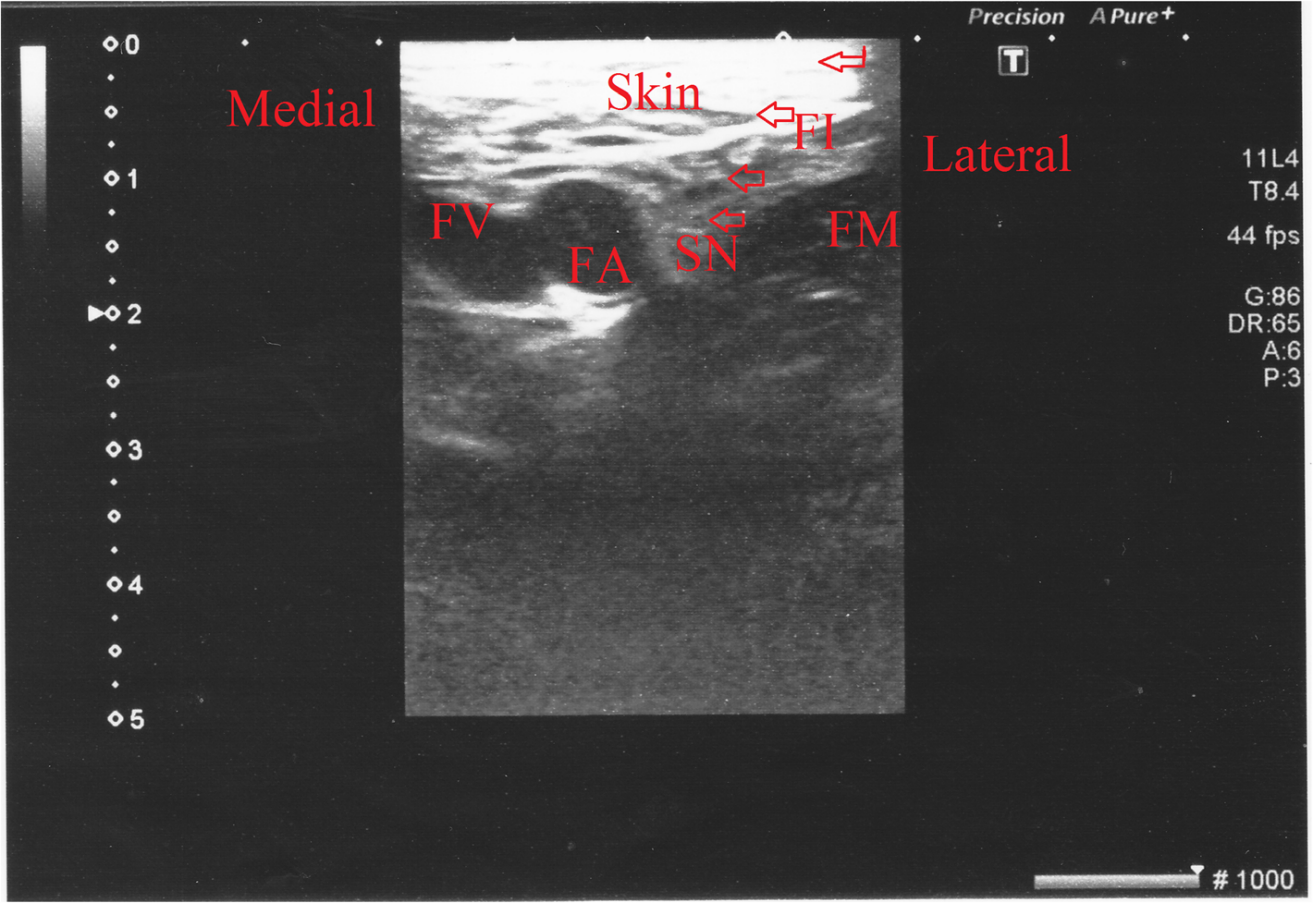
Ultrasound image of the femoral nerve obtained with the anterior approach during the block is shown in the short axis (transverse view). FI: fascia iliaca, IM: liopsoas muscle, LA: local anesthetic, FA: femoral artery, FV: femoral vein, FN: femoral nerve, Arrows: Femoral nerve = needle.

For femoral nerve block, the nerve was visualised with concurrent USG and the needle was oriented to the nerve. When the vastus medialis had contracted, the vastus intermedialis and vastus lateralis muscles were visualised and 20 mL of the above-described local anaesthesia solution was administered. Dissemination of the solution was monitored by USG (linear probe) (Fig. 2).

**Fig. 3:**
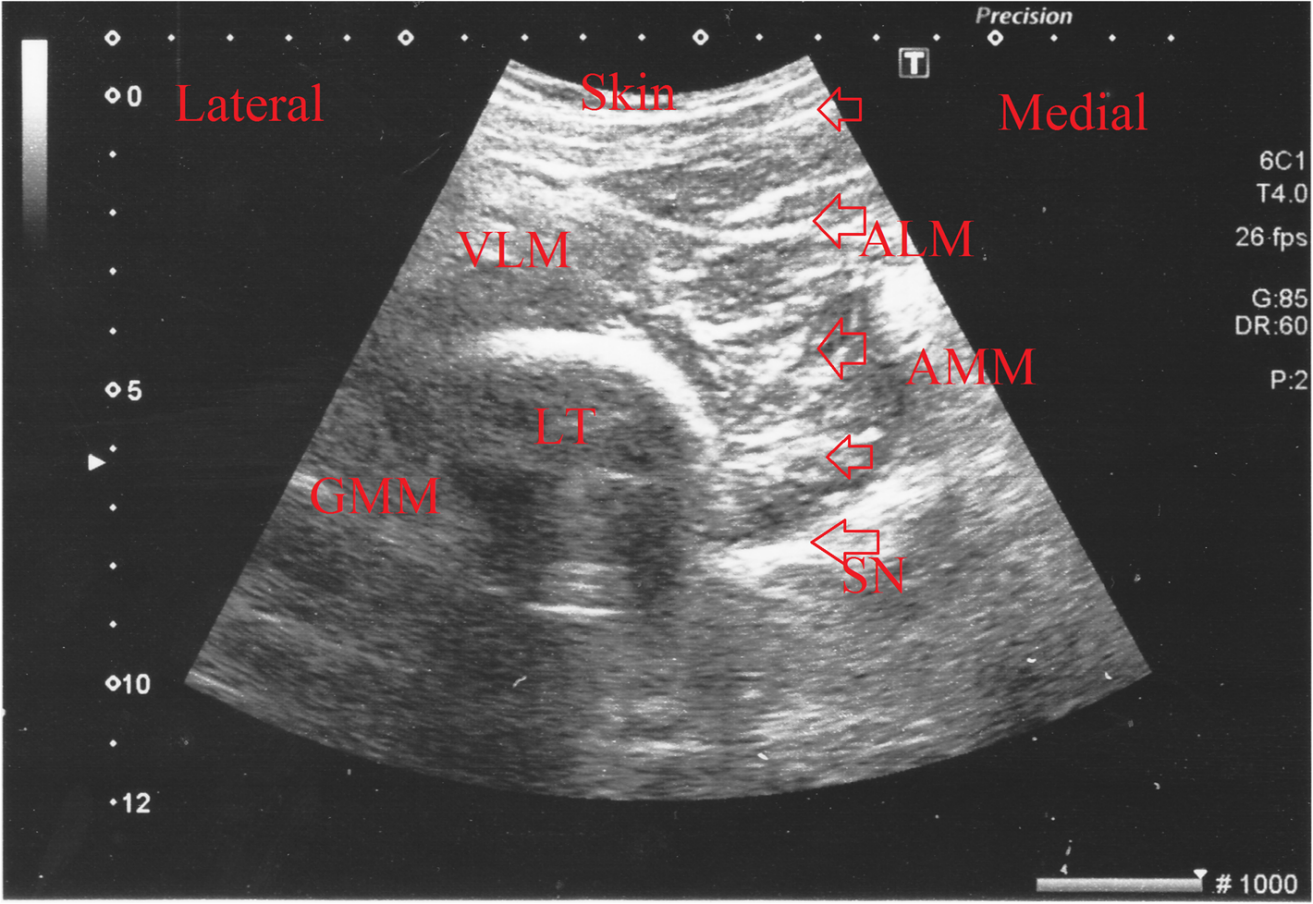
Ultrasound image of the sciatic nerve obtained with the anterior approach during the block is shown in the short axis (transverse view). ALM: adductor longus muscle, AMM: adductor magnus muscle, GMM: gluteus maximus muscle, LT: femur (lesser trochanter), VLM: vastus lateralis muscle, LA: local anesthetic, Arrows: Sciatic nerve; triangles = needle.

For anterior sciatic nerve block, the sciatic nerve was imaged by USG (convex probe) along the needle route and the needle was advanced to the nerve. Following plantar flexion, dorsal flexion, and eversion of the foot, 20 mL of the above-described local anaesthesia solution was administered and its spread was monitored by USG (Fig. 3).

**Fig. 4:**
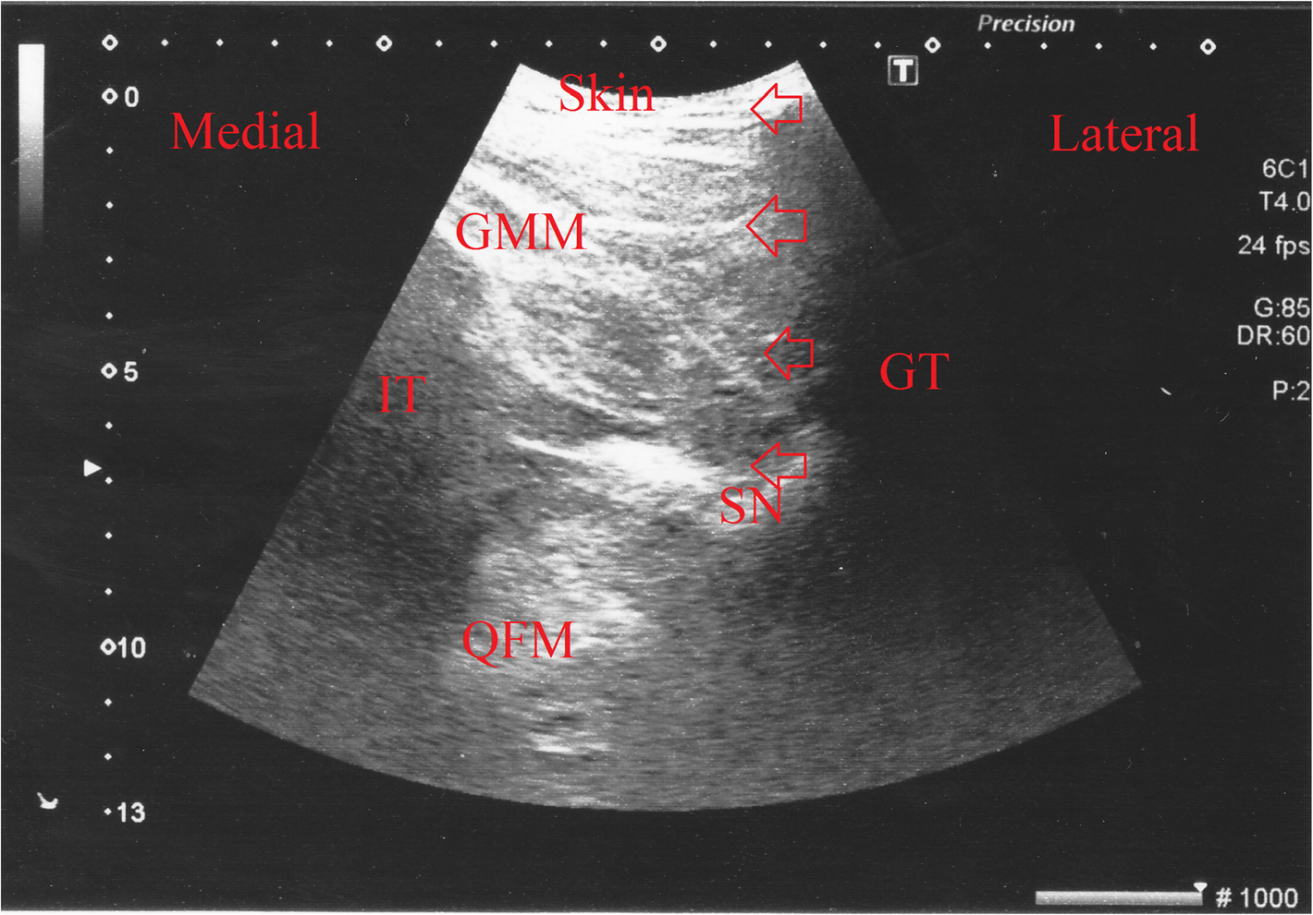
Ultrasound image of the sciatic nerve obtained with the posterior (subgluteal) approach during the block is shown in the short axis (transverse view). GMM: gluteus maximus muscle, GT: greater trochanter, IT: ischial tuberosity, QFM: quatratus femoris muscl, LA: local anesthetic, Arrows: Sciatic nerve; triangles = needle.

For posterior sciatic nerve block, the USG probe was placed between the greater trochanter and the coccyx at the entry point of the needle; the needle was advanced by imaging the nerve. Following plantar flexion, dorsal flexion, and eversion of the foot, 20 mL of the above-described local anaesthesia solution was administered and its spread was monitored by USG (Fig. 4).

After the blocks had been completed, motor block was assessed by monitoring the movement of the ankle joint and knee, while sensory block was assessed by the application of a cold saline bag at 1-min intervals. The block start times were recorded. The time at which the patient no longer perceived cold stimulation of the sciatic and femoral stimulation areas was recorded as the start time of the full femoral-sciatic sensory block; the time at which the patient could not move the knee joint was recorded as the start time of full femoral motor block. The time at which the patient was unable to move the ankle joint was recorded as the start time of full sciatic motor block. After full block had been achieved, a tourniquet was applied to the target extremity and then inflated. Patients underwent surgery at 30 min after administration of the block. Patients with a VAS score ≥ 5 for pain were administered 1 μg fentanyl kg^− 1^ intravenously. The mean fentanyl dose administered per patient after blockade and before surgery was recorded together with the following: mean fentanyl dose per patient after surgery; time to first fentanyl requirement before surgery and after blockade; time to first fentanyl requirement after surgery; time to first diclofenac sodium dose; and total dose of diclofenac sodium administered within the first 24 h postoperatively. Patients with a postoperative VAS score ≥ 5 for pain were also intravenously administered 1 μg fentanyl kg^− 1^.

### Statistical analysis

All data were evaluated using SPSS 11.5 for Windows. Sample size calculation was performed as follows: the required number of participants was determined based on the results of a pilot study that included 10 patients in each group. In that study, the time to sciatic nerve sensory block onset (mean ± standard deviation) was 8.88 ± 4.87 min for group A (anterior approach to sciatic nerve block + femoral nerve block) and 4.70 ± 2.05 min for group P (posterior approach to sciatic nerve block + femoral nerve block). The sample size was calculated as 29 (*n* = 29) for group A and 29 (n = 29) for group P, with α = 5 and 90% power. Thus, 58 patients were enrolled in the present study.

The normality of the data was assessed using the Shapiro–Wilk test. Mean ± standard deviation values were used in parametric tests and median (minimum–maximum) values were used in nonparametric tests. Categorical data are presented as n [5]. An independent samples t-test was used for binary comparisons of group data; the chi-squared test was used for between-group comparisons of categorical data. A *p* value < 0.05 was considered to indicate statistical significance.

## Results

Despite the administration of local anaesthesia, nerve blocks were not achieved in eight patients in group A; these patients were excluded from the study. In addition, the sciatic nerve was not visualised in two patients in group A, and a muscle response was not obtained in response to stimulation; these patients were also excluded, along with three other patients in group A who experienced pain despite an adequate block and administration of high-dose fentanyl. Similarly, four patients in group P were excluded because nerve blocks were not achieved, despite administration of local anaesthesia. Four other patients in group P were excluded from the study because of pain despite an adequate block and administration of high-dose fentanyl. Finally, one patient in group P was excluded because of the development of delirium during the block. No complications occurred in either group during the intraoperative or postoperative periods.

Participant data (e.g., age, height, weight, American Society of Anesthesiologists physical status, tourniquet duration, and surgical duration) are shown in Table 1. There were no statistically significant differences between groups A and P with respect to these data. The start and end times of sciatic nerve and femoral nerve sensory blocks are presented in Table 2. There was a significant difference between groups A and P in terms of the sciatic nerve sensory block start time, which was significantly lower in group P than in group A. In contrast, the sciatic nerve sensory block end time and femoral nerve sensory block start and end times did not significantly differ between the two groups.

**Table 1:** Comparison of demographic characteristics, tourniquet duration, surgical duration, American Society of Anesthesiologists and gender distribution of the groups. (mean ± SD) or (*n*)

		Group A (*n* = 29)	Group P (*n* = 29)	*p*
Age (year)		37.95 ± 12.69	38.95 ± 8.68	0.839
Height (cm)		171.80 ± 14.98	172.30 ± 7.65	0.848
Weight (kg)		78.85 ± 15.22	76.05 ± 11.11	0.511
Tourniquet duration (min)		76.70 ± 32.57	65.55 ± 23.59	0.223
Surgery duration (min)		81.85 ± 30.47	66.35 ± 24.59	0.085
ASA	I (n)	21	22	0.500
	II (n)	8	7	
Gender	Female (n)	22	16	0.083
	Male (n)	7	13	

ASA American Society of Anesthesiologists

**Table 2:** Comparison of sensorial block start and end times after sciatic and femoral nerve block. (mean ± SD)

	Group A (*n* = 29)	Group P (*n* = 29)	*p*
Sensory block start time for sciatic nerve (min)	12.88 ± 4.87	7.70 ± 2.05	^a^0.01
Sensory block end time for sciatic nerve (min)	188.50 ± 69.01	201.85 ± 43.81	0.564
Sensory block start time for femoral nerve (min)	10.39 ± 3.39	9.90 ± 5.49	0.09
Sensory block end time for femoral nerve (min)	146.65 ± 78.67	124.50 ± 17.85	0.074

^a^Statistically significant

The sciatic nerve and femoral nerve motor block start and end times for patients in groups A and P are shown in Table 3; the differences between the two groups were not statistically significant. The results of group comparisons of patient satisfaction, anaesthesia quality, and surgical quality are reported in Table 4. There was a statistically significant difference between the two groups in terms of patient satisfaction, which was significantly higher in group P than in group A. Both anaesthesia quality and surgical quality were also significantly better in group P than in group A.

**Table 3:** Comparison of motor block start and end times after sciatic and femoral nerve block. (mean ± SD)

	Group A (*n* = 29)	Group P (*n* = 29)	*p*
Motor block start time for sciatic nerve (min)	13.55 ± 4.75	10.40 ± 2.13	0.072
Motor block end time for sciatic nerve (min)	115 ± 63.83	109.50 ± 42.17	0.750
Motor block start time for femoral nerve (min)	11.61 ± 4.48	10.16 ± 3.91	0.063
Motor block end time for femoral nerve (min)	99.70 ± 63.83	71.50 ± 18.07	0.061

**Table 4:** Comparison of patient satisfaction, anesthesia quality and surgical quality in groups.(*n*)

					*p*
		2	3	4	
Patient satisfaction	Group A (*n* = 29)	16	9	4	^a^ < 0.01
	Group P (*n* = 29)	4	5	20	
Anesthetic quality	Group A (*n* = 29)	13	7	9	^a^0.005
	Group P (*n* = 29)	3	6	20	
Surgical quality	Group A (*n* = 29)	8	8	13	^a^0.026
	Group P (*n* = 29)	3	3	23	

^a^Statistically significant

Patient satisfaction: 2: moderate, 3: good, 4: excellent

Anaesthesia quality (anaesthesiologist) and surgical quality (surgeon): 1: Failed; general anaesthesia was required, 2: Moderate; complainant, complementary analgesic was needed, 3: Good; little complainant, no need for complementary analgesia, 4: Excellent; patients do not complain

The mean fentanyl dose administered per patient before surgery and after blockade, mean fentanyl dose administered per patient postoperatively, time to the first fentanyl requirement before surgery and after blockade, time to the first fentanyl requirement postoperatively, total dose of diclofenac sodium administered during the postoperative period, and time to the first postoperative diclofenac sodium dose are shown in Table 5. The mean fentanyl dose administered per patient before surgery and after blockade was significantly higher in group P than in group A. After surgery, the mean fentanyl dose administered per patient was significantly lower in group P than in group A. The time to the first fentanyl requirement before surgery and after blockade was significantly shorter in group P than in group A, whereas the time to the first fentanyl requirement after surgery was significantly shorter in group A than in group P. There were no statistically significant differences between the two groups regarding the total dose of diclofenac sodium administered during the first 24 h postoperatively or in the time to first diclofenac sodium dose.

**Table 5:** Comparison of mean fentanyl dose administered per patient after blockade andbefore surgery, mean fentanyl dose administered per patient after surgery, First fentanyl requirement time after blockade and before surgery, First fentanyl requirement time after surgery, total diclofenac sodium amount and first diclofenac sodium requirement times in the postoperative first 24-h periods in the groups. (mean ± SD)

	Group A (*n* = 29)	Group P (*n* = 29)	p
Mean fentanyl dose administered per patient after blockade and before surgery (μg)	00.00 ± 00.00	16.24 ± 7.13	^a^ < 0.01
First fentanyl requirement time after blockade and before surgery (min)	00.00 ± 00.00	4.05 ± 1.47	^a^ < 0.01
Mean fentanyl dose administered per patient after surgery (μg)	147.75 ± 22.30	11.51 ± 2.87	^a^ < 0.01
First fentanyl requirement time after surgery (min)	31.20 ± 17.79	44.03 ± 13.78	^a^ < 0.01
Total amount of diclofenac sodium administered within 24 h (mg)	86.25 ± 34.31	71.25 ± 26.52	0.426
First diclofenac sodium requirement time (min)	332.15 ± 46.50	293.75 ± 77.00	0.646

^a^Statistically significant

## Discussion

In the present study, USG-guided sciatic nerve blocks performed using posterior and anterior approaches were compared in terms of block quality. In both approaches, the block was established using a nerve stimulator to minimise possible complications related to the block technique. In the anterior approach, the sciatic nerve is deeper than in the posterior approach. However, the difficulty involved in sciatic nerve imaging with USG is similar between the anterior approach and other approaches [4]. In addition, a previous study showed that the qualities of sensory and motor blocks achieved with the anterior approach are comparable to those of the posterior approach [4].

In the present study, the results showed a statistically significant difference between anterior and posterior approaches with respect to the start time of the sensory block after completion of the sciatic nerve block, which was earlier in patients treated with the posterior approach (7.70 ± 2.05 min) than in patients treated with the anterior approach (12.88 ± 4.87 min). However, there was no statistically significant difference in the sensory block end time. A previous study [13] demonstrated block start times of 9.42 ± 1.08 min and 7.75 ± 0.97 min using the anterior and posterior approaches, respectively; this difference was significant (*p* = 0.001) and the results were comparable to those of our study.

In contrast, there were no statistically significant differences in terms of the motor block start and end times associated with the anterior and posterior approaches. However, satisfaction was significantly higher in patients who received the posterior approach: 20 of the 29 patients in group P rated their satisfaction as ‘grade 4’ (Table 4). Similarly, the anaesthesia quality in 20 of the 29 patients in group P was graded by the anaesthetist as ‘grade 4’, which demonstrated that significantly higher anaesthesia quality could be achieved using the posterior approach. The surgical quality as assessed by the surgeon was also assigned ‘grade 4’ in 20 of the 29 patients in group P. However, another study [13] found no significant difference in anaesthesia quality between the anterior and posterior methods in terms of either patient- or anaesthesiologist-graded satisfaction. We used questionnaires to evaluate patient satisfaction with a 5-point scale and anaesthesiologist/surgeon satisfaction with a 4-point scale. In our study, the levels of satisfaction reported by the patients, anaesthesiologists, and surgeons were significantly higher in group P than in group A. This indicates a better block quality in group P, despite the initial pain and early fentanyl needs of these patients. In group P, the mean fentanyl dose administered per patient was significantly higher before surgery and after blockade, but significantly lower postoperatively. This can be attributed to the higher quality of the sensory block achieved with the posterior vs. the anterior approach, or to the lower tourniquet-related pain. However, in patients treated using the posterior approach, the time to the first fentanyl requirement before surgery and after blockade was significantly shorter than that in patients treated using the anterior approach; this could be related to the pain associated with the repositioning of the patients’ fractured extremity. Patients who exhibited fracture pain were positioned laterally in group P, in order to place the fractured limb upwards; therefore, fentanyl administration was required earlier, until the block was completed. After surgery, the time to the first fentanyl requirement was significantly longer. In one study [13], a comparison of analgesic needs during the first 24 h postoperatively between patients treated via the anterior approach and those treated via the posterior approach showed no significant differences. This was similar to our study, in which there were no statistically significant differences between groups P and A in terms of both the time to the first analgesic requirement and the total amount of analgesic within the first 24 h postoperatively.

In the anterior approach, the location of the sciatic nerve is significantly deeper than in the posterior approach [4]. In our study, the sciatic nerves of two patients in group A were not imaged with USG, as in the study reported by Ota et al. [4] Nonetheless, we found that there were no differences between the two approaches in terms of sensory and motor block initiation. A previous study revealed that either of the two approaches could be used in patients undergoing minor knee surgery [4]. However, because the posterior femoral cutaneous nerve runs parallel to the sciatic nerve in the gluteal region, sensory block is rarely achieved via the anterior approach. During knee surgery, this is not considered a disadvantage when a tourniquet is used [4]. The fentanyl requirement of patients in that study was similar to that of patients in other studies because most could not tolerate the tourniquet pain [4]. In another study, posterior femoral cutaneous nerve block had no effect on tourniquet pain [14]. Our patients experienced greater tourniquet pain associated with the anterior approach than with the posterior approach; therefore, the total dose of fentanyl administered was significantly higher.

## Conclusion

Sciatic nerve block can be performed using the anterior or posterior approach in patients undergoing surgery for malleolar fracture. However, analgesia should be induced before a posterior block is started, as well as postoperatively, in patients with an anterior block.

## Data Availability

The datasets used and/or analysed during the current study available from the corresponding author on reasonable request.

## Abbreviations

ASA: American Society of Anesthesiologists
SPSS: Statistical Package for the Social Sciences
USG: Ultrasonography
VAS: Visual analog scale
